# Report of Committee of the Wilts Branch of the British Medical Association on County Medical Service

**Published:** 1922-12

**Authors:** Chas. E. S. Flemming


### CORRESPONDENCE.

[Correspondence on all subjects is invited, but we cannot be responsible for the opinions expressed by our correspondents, who must give name and address as a guarantee of good faith, but not necessarily for publication. Correspondents are reminded that conciseness greatly facilitates early insertion.]

### Report of Committee of the Wiltshire Branch of the British Medical Association on County Medical Service.

*To the Editor of* THE HOSPITAL AND HEALTH REVIEW.

SIR,—I was much interested in reading in this month's HOSPITAL AND HEALTH REVIEW Sir Napier Burnett's remarks at the opening of the Insch Cottage Hospital, in which he states that the next move toward a hospital system for each County lies with the medical profession. I thought it would interest you to know that the medical men of Wiltshire have already through the local Branch of the British Medical Association taken steps to co-ordinate and improve the hospital service of the County. A special committee was appointed for this purpose some time ago, and I enclose you a copy of the scheme which was adopted last May by the Branch and the staffs of the various Cottage and Central Hospitals :

1. The County should be divided into three areas, one for the South, one for the West, and one for the North, and the local or primary hospitals in the respective areas should be associated with a General Hospital. This association, whatever form it might take, would be entirely voluntary, and would not involve any sort of control or supervision. The General Hospital for the South would be Salisbury Infirmary; for the West, Royal United Hospital, Bath; and for the North, a hospital in Swindon.

2. It is essential to the satisfactory working of any hospital service that there should be some system under which a medical practitioner sending a patient to a hospital should at the same time send a record of the case, and when the patient leaves the hospital a record of diagnosis and of treatment given and required should be sent to the practitioner to whose care the patient will pass.

3. It would much relieve the pressure on the beds of the Central and Cottage Hospitals if more adequate convalescent accommodation could be provided. Whether these beds should be associated with the several hospitals or in a central convalescent home, is a question that requires further consideration.

4. Arrangements should if possible be made with the staff of the General Hospital to visit cases in a local hospital at the request, and only at the request, of a member of the staff of that hospital. The method of selecting the consultant is a matter that must rest with the medical attendant of the patient, and the medical board of the hospital concerned. Similar arrangements might be made with any special hospital.

5. If possible, arrangements should be made with the local authority for the various communal services, treatment of school children, T.B. and V.D. clinics, maternity and child welfare clinics, maternity beds, all to be conducted where the conditions are suitable in the local hospital, under the general supervision of the medical officer of the authority concerned, the staff of the hospital so far as possible giving the necessary attendance, arrangements being made by the local authority by which the attendance of a specialist may be obtained when necessary.

6. There is an urgent necessity for the establishment of a pathological and bacteriological laboratory, so situated that its services would be available for all the hospitals, health authorities and medical practitioners in the County. It was hoped that it might be possible to get the services of a pathologist with a properly equipped laboratory available for the whole County, but various difficulties arose, and meanwhile Salisbury Infirmary and the Royal United Hospital, Bath, have instituted pathological laboratories, which are now being equipped and will, it is expected, be opened before long. They will be under the management of first-class pathologists, and will undertake pathological, bacteriological, bio-chemical and chemical examination and research, and it is hoped that arrangements may be made for a member of the staff of either laboratory to visit any part of the County where his services may be required; and patients can be sent to the laboratories, where medical men themselves will always be welcome visitors.

The success of any such an undertaking must be largely dependent upon the support given by private practitioners, by the hospitals and the local health authorities.

Yours faithfully,

CHAS. E. S. FLEMMING, M.R.C.S., L.R.C.P.

